# Accuracy and inter-observer variability of 3D versus 4D cone-beam CT based image-guidance in SBRT for lung tumors

**DOI:** 10.1186/1748-717X-7-81

**Published:** 2012-06-08

**Authors:** Reinhart A Sweeney, Benedikt Seubert, Silke Stark, Vanessa Homann, Gerd Müller, Michael Flentje, Matthias Guckenberger

**Affiliations:** 1 Department of Radiation Oncology, University of Wuerzburg, Josef-Schneider-Str, 11 97080 Wuerzburg, Germany

**Keywords:** Lung cancer, Image-guidance, Cone-beam CT, Inter-observer variability, Respiration correlated imaging

## Abstract

**Background:**

To analyze the accuracy and inter-observer variability of image-guidance (IG) using 3D or 4D cone-beam CT (CBCT) technology in stereotactic body radiotherapy (SBRT) for lung tumors.

**Materials and methods:**

Twenty-one consecutive patients treated with image-guided SBRT for primary and secondary lung tumors were basis for this study. A respiration correlated 4D-CT and planning contours served as reference for all IG techniques. Three IG techniques were performed independently by three radiation oncologists (ROs) and three radiotherapy technicians (RTTs). Image-guidance using respiration correlated 4D-CBCT (IG-4D) with automatic registration of the planning 4D-CT and the verification 4D-CBCT was considered gold-standard. Results were compared with two IG techniques using 3D-CBCT: 1) manual registration of the planning internal target volume (ITV) contour and the motion blurred tumor in the 3D-CBCT (IG-ITV); 2) automatic registration of the planning reference CT image and the verification 3D-CBCT (IG-3D). Image quality of 3D-CBCT and 4D-CBCT images was scored on a scale of 1–3, with 1 being best and 3 being worst quality for visual verification of the IGRT results.

**Results:**

Image quality was scored significantly worse for 3D-CBCT compared to 4D-CBCT: the worst score of 3 was given in 19 % and 7.1 % observations, respectively. Significant differences in target localization were observed between 4D-CBCT and 3D-CBCT based IG: compared to the reference of IG-4D, tumor positions differed by 1.9 mm ± 0.9 mm (3D vector) on average using IG-ITV and by 3.6 mm ± 3.2 mm using IG-3D; results of IG-ITV were significantly closer to the reference IG-4D compared to IG-3D. Differences between the 4D-CBCT and 3D-CBCT techniques increased significantly with larger motion amplitude of the tumor; analogously, differences increased with worse 3D-CBCT image quality scores. Inter-observer variability was largest in SI direction and was significantly larger in IG using 3D-CBCT compared to 4D-CBCT: 0.6 mm versus 1.5 mm (one standard deviation). Inter-observer variability was not different between the three ROs compared to the three RTTs.

**Conclusions:**

Respiration correlated 4D-CBCT improves the accuracy of image-guidance by more precise target localization in the presence of breathing induced target motion and by reduced inter-observer variability.

## Background

Breathing induced motion of tumors and organs-at-risk are significant sources of uncertainties in radiotherapy of pulmonary and abdominal targets [1], which affects the accuracy at all stages of the treatment process: target definition, safety margin selection, dose calculation, patient set-up and treatment delivery. Respiration correlated CT (4D-CT) imaging is considered as method of choice for treatment planning in the thoracic and abdominal region: 4D-CT reduces motion artifacts for precise target volume delineation and simultaneously allows patient-individual motion assessment for adjustment of safety margins [2,3]. It has been shown that image-guidance (IG) is most important to improve the overall accuracy of lung cancer treatment [4]; consequently, respiration correlated 4D-CT needs to be integrated into a consistent 4D IG work-flow [5].

Respiration correlated cone-beam CT (4D-CBCT) has been commercialized recently [6] and allows the realization of a volumetric 4D image guidance workflow. However, it remains unclear whether 4D-CBCT actually improves the accuracy of IG compared to conventional 3D-CBCT. Phantom studies have indicated similar accuracy of IG using 3D-CBCT and 4D-CBCT [7]. Additionally, IG using 4D-CBCT may be associated with potential disadvantages; image acquisition of a respiration correlated 4D-CBCT takes longer than 3D-CBCT, which affects patient through-put and may increase the risk of patient motion between imaging and treatment. Respiration correlated imaging at treatment delivery also increases the complexity of IG, which may introduce additional uncertainties in clinical practice. Finally, modern and cost-intense technologies are being discussed controversially especially in situations where clinical evidence is scarce.

Several centers have reported their experiences with IG using 3D-CBCT or 4D-CBCT and various techniques and work-flows have been used [8-15]. Therefore, it was the aim of this study to compare the accuracy of 3D-CBCT and 4D-CBCT based IG techniques. In order to have clinically representative results, only commercially available soft- and hardware was used and no research equipment was allowed. Additionally, all IG techniques were performed independently by three experienced radiation oncologists (RO) and by three experienced radiotherapy technicians (RTT) to evaluate inter-observer variability.

## Methods

This retrospective simulation study is based on 21 consecutive patients, who were treated with 4D-CBCT based image-guided SBRT for early stage primary non-small cell lung cancer (NSCLC) or pulmonary metastases; target characteristics are described in Table 1.

### Clinical treatment planning and delivery

A respiration-correlated 4D-CT was acquired with a 24-slice helical CT scanner (Somatom Sensation Open; Siemens Medical Solutions, Erlangen, Germany). A pressure sensor placed in an elastic belt around the abdomen generated the external breathing signal (Anzai AZ-733 V; Anzai Medical Solutions, Japan). Two 4D-CT series reconstructed at end-inhalation and end-exhalation phases were used for treatment planning in the Pinnacle treatment planning system (Philips Radiation Oncology Systems, Milpitas, CA, USA). The macroscopic tumor was delineated in the end-exhalation phase, where breathing motion and motion artefacts are expected to be smallest [1]. No margin was added for generation of the clinical target volume (CTV). The structure of the CTV was converted into a 3D mesh, propagated into the end-inhalation phase where the position of the CTV was adjusted. The internal target volume (ITV) was generated based on the CTV contours in end-exhalation and end-inhalation and the planning target volume (PTV) was generated with a safety margin of 5 mm [4,16].

Treatment plans were generated for an Elekta Synergy S^TM^ linear accelerator equipped with cone-beam CT technology (Elekta, Crawley, UK). The 4D-CT series in end-exhalation and all planning contours were transferred as planning reference into the XVI^TM^ image-guidance software, version 4.5 (Elekta, Crawley, UK). At treatment delivery, a respiration correlated 4D-CBCT was acquired using the standard parameters provided by the manufacturer for image acquisition and reconstruction (200° rotation for acquisition of 1320 frames within 4 minutes, 20 mA and 16 ms per frame, 120 kV, S20 filter).

### Image-quality of 3D-CBCT and 4D-CBCT

Image quality of the 3D-CBCT and the 4D-CBCT was scored by six observes: three ROs and three RTTs. All observes had >2 years clinical experience in image-guided SBRT for lung tumors and had been trained by the manufacturer and by MG in the use of the XVI^TM^ 4.5 system. The criterion for image-quality scoring was visibility of the pulmonary target for precise manual verification of the IG results. Score 1 was defined as clearly visible tumor without any difficulties in manual verification of IG results; score 2 was defined as visible tumor with difficulties in manual verification of IG results; score 3 was defined as image quality, where precise visual localization of the target is hardly or not possible for manual verification of IG results.

### Image-guidance protocols

Three different IG techniques were evaluated independently by all six observers; other observer`s and the clinical results were made unavailable for all observers. The three IG techniques were performed in the following sequence.

#### Image-guidance using manual registration of the planning ITV contour and the verification 3D-CBCT (IG-ITV)

This was the standard IG technique at our department prior to the introduction of the 4D-CBCT [8] and has been described as routine practice by other institutions [9-11]. The rectangular clipbox for automatic registration of the planning reference CT and the verification CBCT in the XVI^TM^ software was confined to the vertebral spine on the level of the pulmonary tumor for evaluation of patient set-up. The 4D-CBCT was visualized as a conventional “slow” 3D-CBCT, which was the average intensity projection (AIP) of all 4D-CBCT phases. The contours of the ITV and PTV were projected onto the AIP 3D-CBCT and their position was adjusted manually in all three planes to the motion-blurred tumor.

#### Image-guidance using automatic registration of planning 4D-CT and the verification 4D-CBCT (IG-4D)

Using respiration correlated 4D-CBCT for IG is our current standard of practice and simultaneously the proposed technique by the manufacturer and other institutions [12,13]. After bony registration as described above, a so-called mask was generated for automatic image registration of the target: only the volume of the reference planning CT within this mask is used by the XVI^TM^ software for automatic soft-tissue registration. Generation of this mask was done independently by all observes via expansion of the CTV with a 2-3 mm margin and manual exclusion of all bony structures using a drawing tool (ribs, vertebrae, sternum). Automatic soft-tissue registration between the volume of the reference planning 4D-CT (end-exhalation phase) inside this mask and all ten phases of the respiration correlated 4D-CBCT was performed: the position of the target was identified in each breathing phase. The target position in the end-exhalation planning 4D-CT phase relative to the target position in the end-exhalation 4D-CBCT phase was then calculated as the tumor position error. Manual adjustment of the registration was allowed at the discretion of the observer.

#### Image-guidance using automatic registration of the planning 4D-CT and the verification 3D-CBCT (IG-3D)

This work-flow has been described by several institutions in literature [14,15]. Bony registration was performed initially and a mask for soft-tissue registration was defined as described above. Automatic registration was then performed between the planning end-exhalation 4D-CT series as reference and the verification AIP 3D-CBCT. No manual adjustment was allowed.

### Statistical analysis

Statistica X was utilized for statistical analysis (Statsoft, Tulsa, OK, USA). Mann–Whitney-*U* test was performed for comparison of two subset analyses and Wilcoxon test was used for matched pair analyses. Chi-squared test was used for categorical variables. Differences were considered significant for p < 0.05.

## Results

### Target visualization in 3D-CBCT and 4D-CBCT images

**Figure 1:**
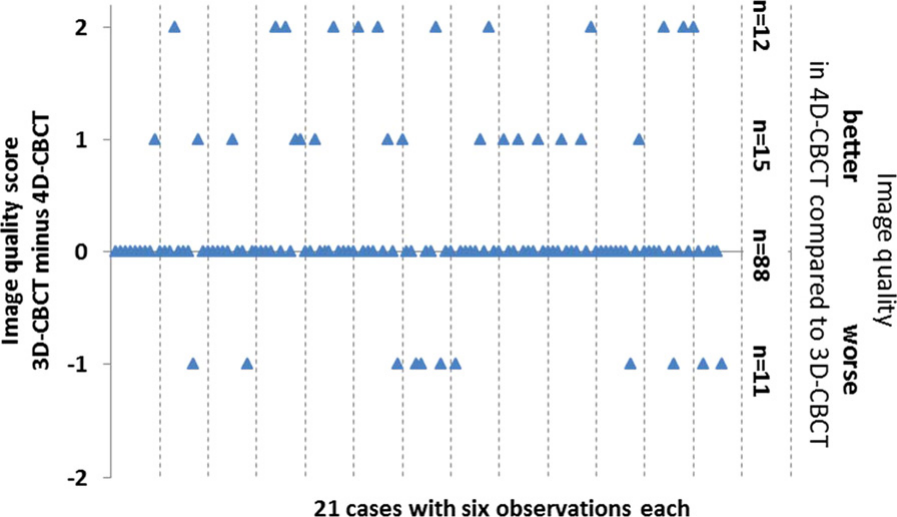
Comparison of image-quality in 3D-CBCT and 4D-CBCT for all 21 cases and 6 observers individually.

The image quality scores for visual identification of the target position and verification of IG results were 1.5 ± 0.8 and 1.3 ± 0.4 averaged over all six observes and 21 cases for 3D-CBCT and 4D-CBCT respectively; this difference was not statistically significant (p = 0.42). However, significantly different image quality scores were observed for 3D-CBCT compared to 4D-CBCT when all 252 observations were considered individually. The worst image quality score of 3 was given in 19 % (24/126) and 7.1 % (9/126) observations for 3D-CBCT and 4D-CBCT (p = 0.005), respectively; simultaneously, the best score of 1 was given in 67 % (84/126) and 77 % (97/126) observations using 3D-CBCT and 4D-CBCT, respectively (p = 0.02). Differences in the image quality score between 4D-CBCT and 3D-CBCT for all 126 observations are shown in Figure 1.

Image quality scores were not significantly different between ROs and RTTs: averaged values were identical with 1.4 ± 0.7 and the worst score of 3 was given for 14 % and 12 % observations in the RO and RTT group (p = 0.57), respectively. Both RTTs and ROs scored the worst image quality of 3 significantly less frequently and the best score of 1 significantly more frequently in 4D-CBCT images compared to 3D-CBCT images.

### Differences in target positions between IG technologies

Patient set-up errors, absolute tumor position errors and tumor base-line shifts relative to the bony anatomy are summarized in Table 2.

**Table 1:** Target characteristics; central or peripheral location is based on the RTOG definition

	**# of cases**	**Median**	**Range**
**Upper/middle/lower lobe [n]**	7/2/12		
**Central/peripheral location [n]**	2/19		
**Maximum GTV diameter [cm]**		1.9	0.6 – 3.9
**3D motion amplitude [mm]**		6.3	1.3 – 17

**Table 2:** 3D set-up errors (bone registration), tumor position errors (based on IG-4D) and tumor base-line shifts relative to the bony anatomy

	**Average**	**Standard deviation**	**Minimum**	**Maximum**
**3D Set-up errors [mm]**	7.0	±2.4	2.3	10.7
**3D Tumor position errors [mm]**	8.6	±4.4	2.3	20.9
**3D Tumor base-line shifts [mm]**	4.9	±4.9	2.0	18.9

IG-4D using respiration correlated 4D-CBCT served as gold-standard for comparison with the two IG techniques using 3D-CBCT. Averaged results were calculated for all six observers and differences between 4D-CBCT and 3D-CBCT based IG are summarized in Table 3. Systematic differences of the tumor position between 4D-CBCT and 3D-CBCT based IG were <1 mm in all directions except a systematic difference of 1.2 mm in SI direction between IG-4D and IG-3D. Random variability between 4D-CBCT and 3D-CBCT based IG expressed as one standard deviation was <1 mm in LR direction and <2 mm in AP direction (p = 0.004). Variability was largest in SI with 1.5 mm and 4.3 mm for IG-4D vs. IG-ITV and IG-4D vs. IG-3D (p = 0.02), respectively. Differences of the tumor position as a 3D error vector were 1.9 mm ± 0.9 mm and 3.6 mm ± 3.2 mm for IG-4D vs. IG-ITV and IG-4D vs. IG-3D, respectively, and the difference between the two 3D-CBCT techniques in comparison to 4D-CBCT was statistically significant (p < 0.01).

**Table 3:** Differences between the IG technique using 4D-CBCT and the two IG techniques using 3D-CBCT (average ± one standard deviation [StDev])

								
	**LR [mm]**		**SI [mm]**		**AP [mm]**		**3D vector [mm]**	
	**Average**	**St Dev**	**Average**	**St Dev**	**Average**	**St Dev**	**Average**	**St Dev**
**IG-4D minus IG-3D**	0.1	± 0.9	1.2	± 4.3	0.0	± 1.8	3.6	± 3.2
**IG-4D minus IG-ITV**	0.0	± 0.7	0.9	± 1.5	0.0	± 1.0	1.9	± 0.9

Left-right direction (LR), superior-inferior direction (SI) and anterior-posterior direction (AP).

There was one outlier where IG-3D resulted in a very large difference in the tumor position compared IG-4D of 15 mm. Average image quality score of the 4D-CBCT and 3D-CBCT was 1.8 and 2.8, respectively, indicating that automatic image registration in IG-3D failed because of a poorly visualized tumor position. The difference between IG-4D and IG-ITV was only 1.3 mm for that case indicating the manual registration was more effectively coping with the suboptimal image quality of the 3D-CBCT.

**Figure 2:**
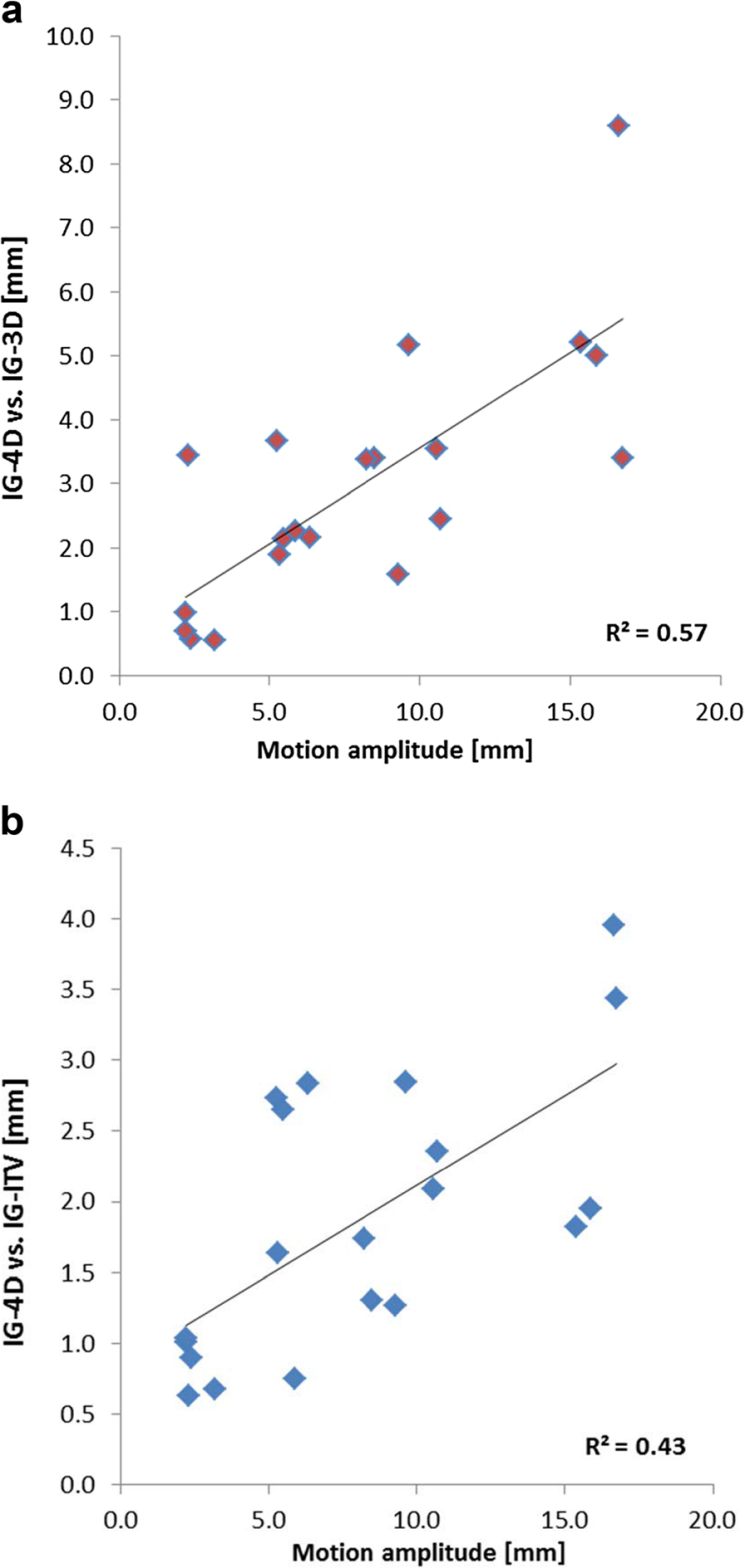
Correlation between the motion magnitude of the pulmonary target and differences between the image-guidance techniques using 4D-CBCT (IG-4D) and 3D-CBCT (IG-ITV and IG-3D).

After exclusion of this outlier, a significant correlation in linear regression analysis between the motion amplitude of the tumor and differences between the 4D-CBCT and 3D-CBCT IG techniques was observed (Figure 2): IG-4D vs. IG-3D (r^2^ = 0.57; p = 0.001) and IG-4D vs. IG-ITV (r^2^ = 0.43; p = 0.002). Increased motion amplitude resulted in increased discrepancies between 4D-CBCT and 3D-CBCT IG techniques.

Absolute differences of the tumor position between IG-4D and IG-ITV were significantly correlated with the image quality score of the 3D-CBCT (p < 0.01): the absolute difference in SI was 2.1 ± 1.7 mm, 2.8 mm ± 2.9 mm and 5.8 mm ± 5.7 mm for an image quality score of 1, 2 and 3 (p < 0.01), respectively. This correlation was of borderline significance for IG-3D (p = 0.05) and no such correlation was observed for image quality scores of the 4D-CBCT.

Averaged results of the IG techniques were calculated separately for ROs and RTTs. The 3D difference of the target position between ROs and RTTs was 0.6 mm ± 0.8 mm using the IG-4D technique and 1.6 mm ± 0.9 mm using the IG-ITV technique (p < 0.001). Identical results were obtained by the ROs and RTTs using IG-3D, where no manual adjustment of the automatic image registration was allowed.

### Inter-observer variability of the IG technologies

Inter-observer variability was calculated as one standard deviation between the six observers and as maximum range between the six observers (Table 4). For IG-3D, where no manual adjustment of the automatic image registration results was allowed, variability between the six observers was <1 mm in all cases (detailed results not shown). Inter-observer variability was significantly larger for IG-ITV compared to IG-4D: variability as one standard deviation was 1.5 mm and 0.6 mm in SI direction (p = 0.002) and the maximum range between the six observers was 3.8 mm and 1.8 mm on average, respectively.

**Table 4:** Inter-observer variability for IG-4D and IG-ITV separately in left-right direction (LR), superior-inferior direction (SI) and anterior-posterior direction (AP)

							
		**Variability as standard deviation between observers**			**Variability as maximum range between observers**		
		**LR[mm]**	**SI[mm]**	**AP[mm]**	**LR[mm]**	**SI[mm]**	**AP[mm]**
**IG-4D**	All observers	0.3	0.6	0.3	0.9	1.8	0.7
	ROs	0.3	0.5	0.2	0.6	0.9	0.4
	RTTS	0.3	0.4	0.3	0.5	0.8	0.6
**IG-ITV**	All observers	0.8	1.5	1.0	2.2	3.8	2.8
	ROs	0.8	1.6	0.9	1.6	3.1	1.7
	RTTS	0.6	1.2	0.9	1.1	2.2	1.8

Inter-observer variability is shown for all six observers and separately for the three ROs and three RTTs.

**Figure 3:**
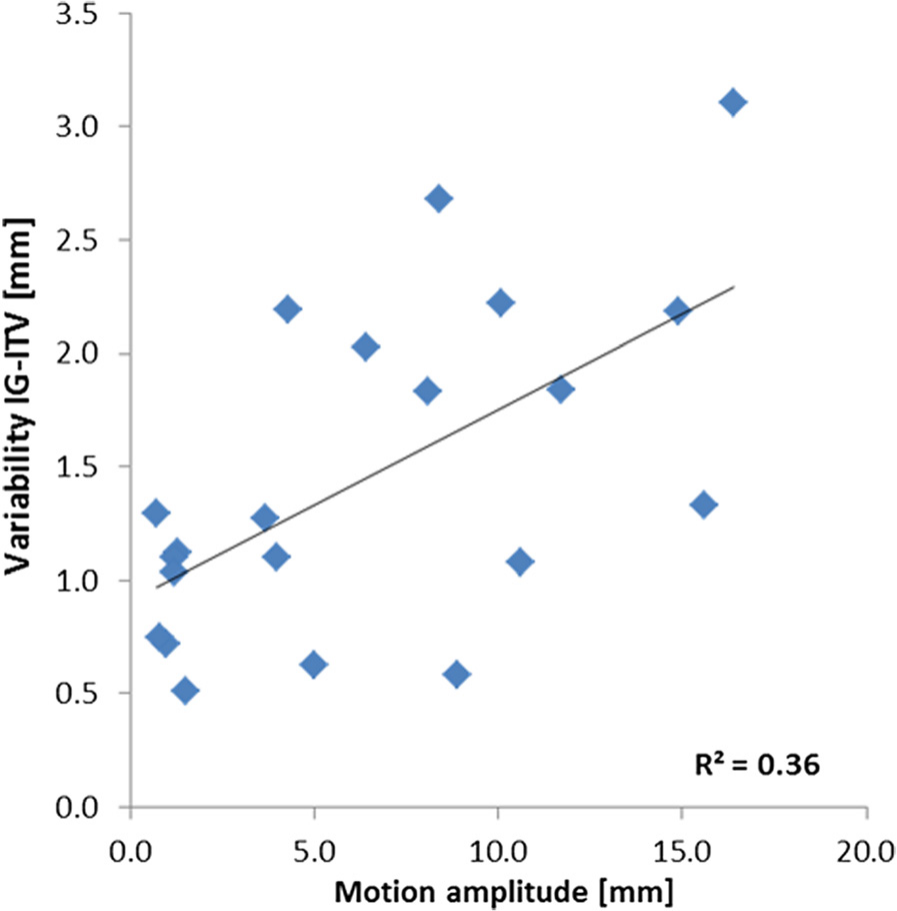
Correlation between the motion magnitude of the pulmonary target and inter-observer variability using the IG-ITV technique.

For IG-ITV there was a significant correlation in linear regression analysis between inter-observer variability in SI direction and motion amplitude of the target (r^2^ = 0.36; p < 0.001) (Figure 3): inter-observer variability was larger in mobile tumors. Such a correlation was not observed for IG-4D. Inter-observer variability was not significantly correlated with the image quality scores.

Inter-observer variability was analyzed separately among ROs and RTTs and no differences were observed.

## Discussion

Image-guidance is considered a prerequisite for most accurate delivery of SBRT [17,18] and cone-beam CT is one of the most frequently used in-room imaging technologies. However, the details of CBCT based image-guidance are poorly defined. Phantom studies have suggested that 3D-CBCT might result in equivalent accuracy of IG compared to 4D-CBCT because the “slow-CT” character of the 3D-CBCT contains all necessary motion information for consistent integration of breathing motion into IG.

Wang et al. evaluated the accuracy of matching the planning ITV contour to the motion blurred target and an accuracy of 1 mm was described in that phantom study. Accuracy in clinical patient treatment was not evaluated. This IG-ITV technique has been practiced by several institutions for lung [8-11] and liver tumors [19], where feasibility in routine practice was described.

Hugo et al. performed a study where two image-guidance techniques were compared: 1) registration of a planning slow-CT scan and a verification 3D-CBCT (IG-3D) and 2) registration of a planning 4D-CT scan with a verification 4D-CBCT (IG-4D) [7]. Similar accuracy was described in the phantom part of the study. In the clinical part based on eight patients, the differences between the two techniques were about 1 mm without an influence of the motion magnitude on the accuracy of both techniques. Automatic registration of the planning CT and the verification 3D-CBCT has been reported by other clinical studies [14,15]; however the details of the IG work-flow were not provided.

**Figure 4:**
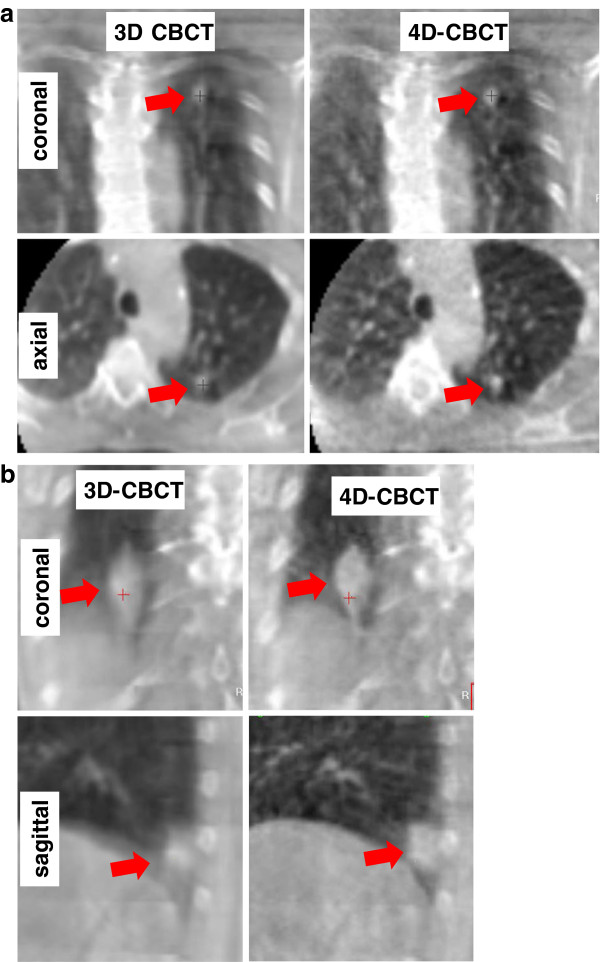
**Examples of image quality in 3D-CBCT and 4D-CBCT. **The arrow indicates the position of the target. **a**) Small target in the upper lobe with a 3D motion amplitude of 9.3 mm, where averaged image quality was 3.0 and 1.8 in 3D-CBCT and 4D-CBCT, respectively. **b**) Target in the lower lobe immediately superior to the diaphragm with a 3D motion amplitude of 16.6 mm, where averaged image quality was 3.0 and 1.3 in 3D-CBCT and 4D-CBCT, respectively.

In contrast to phantom studies describing no clinically relevant potential of 4D-CBCT to improve the accuracy of IG, our study based on 21 consecutive lung cancer patients does not support this conclusion. Six observers described improved visualization of the pulmonary targets in 4D-CBCT compared to 3D-CBCT, which is essential for precise verification of the image-guidance procedure by the ROs or RTTs. Two situations were identified where 4D-CBCT was especially superior to 3D-CBCT (Figure 4): 1) small tumors with large motion amplitude and 2) tumors located immediately superior to the diaphragm, where motion blurring made separation of the tumor from the diaphragm difficult.

Differences between IG using 4D-CBCT as gold standard and the two IG techniques using 3D-CBCT were 3.6 mm (IG-3D) and 1.9 mm (IG-ITV) on average. These uncertainties of 3D CBCT IG appear especially large when compared to the average base-line shift of 4.9 mm in our study, the reason for performing soft-tissue IG. Korreman et al. estimated the residual uncertainty of the IG procedure to 20 % of the initial motion [5], which is optimistic based on our results. Differences in the tumor position between 4D-CBCT and 3D-CBCT based IG increased with increasing motion magnitude of the pulmonary targets and increased with worse image quality scores of 3D-CBCT. These results clearly indicate that 3D-CBCT is not fully sufficient for full motion integration into IG.

This finding of improved accuracy using 4D-CBCT compared to 3D-CBCT is in contrast to the study by Hugo et al. [7], which could be explained by two reasons. First, our study is based on a larger number of patients and poor image quality of the 3D-CBCT with larger uncertainties of IG was observed especially in small and mobile tumors and in tumors located immediately superior to the diaphragm. Detailed information about the tumor size and location was not provided by Hugo et al. such that it is unknown whether these patients “at risk” for decreased accuracy of IG using 3D-CBCT were represented in that study.

Second, we used the end-exhalation CT phase as planning reference for the IG-3D technique, because this phase should resemble most closely the 3D-CBCT: the tumor remains in the exhalation phase of the breathing cycle for the longest time [20] resulting in highest pixel intensities in the exhalation position of the 3D-CBCT. Based on the study by Hugo et al., a slow-CT or AIP as planning reference might improve the accuracy of 3D-CBCT based image guidance. However, acquisition of a planning slow-CT or reconstruction of an AIP was not possible using the Siemens 4D-CT scanner nor the Pinnacle treatment planning system and research software was not allowed in our study protocol. This was done to make results more representative for daily clinical practice outside of specialized academic departments.

In addition to the lower accuracy of 3D-CBCT based IG, clinically relevant inter-observer variability of IG-ITV was observed. Variability in SI direction expressed as one standard deviation between the six observers was 1.5 mm and the range between the six observers was 3.8 mm on average. This inter-observer variability of IG-ITV was correlated with the motion magnitude of the tumor, which highlights the difficulties of precise target localization using the motion blurred 3D-CBCT images. In contrast, inter-observer variability was substantially smaller for 4D-CBCT based IG and was not correlated with the motion amplitude of the target.

It was interesting to see very small differences between ROs and RTTs. Close agreement was observed in scoring the image quality of 3D-CBCT and 4D-CBCT: the image-quality improvement of 4D-CBCT compared to 3D-CBCT was of similar magnitude between both groups. Differences in image-guided target localization between RTTs and ROs were <1 mm for 4D-CBCT based IG-4D and <2 mm for 3D-CBCT based IG-ITV. Inter-observer variability was also not significantly different between ROs and RTTs.

To the best of our knowledge, there is no data in literature about patterns of practice of image-guidance with detailed description of the responsibilities of the different professional groups. An expert group of the European Society of Therapeutic Radiology and Oncology–European Institute of Radiotherapy (ESTRO–EIR) provided a detailed guideline about volumetric IGRT, which emphasized the importance of visual verification of the IGRT results; however, no responsibilities were described most likely because of the legal diversity in Europa [21]. Guidelines by the American Society for Therapeutic Radiology and Oncology (ASTRO) and the American College of Radiology (ACR) state that IGRT images need to be “reviewed by the physician initially and then periodically” during the treatment course; whether this review process takes place online prior to the treatment or offline after treatment delivery remains open [22].

Based on the results of our study combined with the German regulations and international guidelines, we changed our standard operation procedures for cone-beam CT based image-guidance in pulmonary SBRT. 4D-CBCT is the imaging modality of choice and 3D-CBCT is only used for verification after the IGRT couch shift and after treatment delivery. The IGRT process is reviewed online by the radiation oncologist prior to delivery of the first radiotherapy treatment fraction. At consecutive fractions, the IGRT process is performed by the RTTs and reviewed offline by the ROs; online review of all IG results by the ROs prior to each treatment fraction had been our standard of practice before. In cases of base-line shifts >1 cm, the responsible RO is informed for immediate review because of potential overdosage of critical organs at risk [23].

## Conclusions

Respiration correlated 4D-CBCT improved the accuracy of image-guidance by more precise target localization in the presence of breathing induced target motion and by reduced inter-observer variability compared to 3D-CBCT. Respiration correlated 4D-CBCT is therefore the recommended volumetric IG technology in SBRT for lung tumors. No differences in IG accuracy and reproducibility were observed between ROs and RTTs. Depending on national regulations and national guidelines, the results of this study offer the possibility to actively and responsibly define the role of specifically trained RTTs in the IGRT process for pulmonary SBRT.

## Competing interests

There are no competing interests for this study.

## Authors’ contributions

MG designed the study, participated in the data collection and performed the data analysis. BS, SS, VH, GM, RAS and MF participated in data collection. All authors performed critical review of the manuscript and finally approved the manuscript.
